# Honour and subsistence: invention, credit and surgery in the nineteenth century

**DOI:** 10.1017/S0007087416001126

**Published:** 2016-12

**Authors:** SALLY FRAMPTON

**Affiliations:** * 2nd Floor, Gibson Building, Radcliffe Observatory Quarter, University of Oxford, Woodstock Road, Oxford, OX2 6GG, UK. Email: Sally.frampton@ell.ox.ac.uk.

## Abstract

The origins of contemporary exclusion of surgical methods from patenting lie in the complexities of managing credit claims in operative surgery, recognized in the nineteenth century. While surgical methods were not deemed patentable, surgeons were nevertheless embedded within patent culture. In an atmosphere of heightened awareness about the importance of ‘inventors’, how surgeons should be recognized and rewarded for their inventions was an important question. I examine an episode during the 1840s which seemed to concretize the inapplicability of patents to surgical practice, before looking at alternatives to patenting, used by surgeons to gain social and financial credit for inventions.

## Introduction

According to the 2013 guidelines of the United Kingdom Intellectual Property Office, ‘methods of treatment by therapy or surgery or methods of diagnosis performed directly on the human or animal body are unpatentable’.^1^ This legislation aligns with that of the European Patent Office, which similarly excludes from patenting any therapeutic practice which involves physical intervention upon the human or animal body. In the USA, medical and surgical methods may be patented but patentees cannot sue for infringement, meaning that other practitioners are free to use the method without fear of legal or financial repercussions.^2^ In fact, while nations differ somewhat in how they legally enshrine it, the exclusion of methods of treatment is a global trend, with the vast majority of countries following similar patterns of patent law.^3^ This is because surgical patents are perceived to dramatically limit the freedom of medical professionals to take advantage of best practice.

This legal framework sets in place a fundamental division between methods and material products in medicine: the non-patentability of procedures lies in contrast to instruments, devices and pharmaceutical compositions, all of which can be subject to patent protection. This exclusion of methods has implications for all medical specialities, most of which incorporate procedures such as sample collecting and interventional diagnostic testing into their practice. But it is for surgeons that the implications are greatest. While drugs and instruments play a vital role in its practice, surgery is premised upon methods of physical intervention upon the human body. As such, the locus of surgery –the operative technique – remains steadfastly unpatentable, making the intellectual property of surgery a rather different matter to that of other medical disciplines. In a legal climate where high-profile cases such as that of Myriad Genetics, the diagnostics company who attempted to patent the BRCA genes, demonstrate the expanse and complexity of current patent law in medicine, the relative imperviousness of the surgical sphere from patenting stands out.^4^ This distinction made between manufactured compositions and methods is well known and often discussed among scholars of patent law. But a historical perspective can help lead us to a markedly better understanding of how and why it came about and its long-standing implications for the surgical community.

In this paper I seek to historicize the non-patentability of surgical methods and the attendant issues of physical intervention, standardization, profitability and professional ethics which governed it. In Britain, I argue, the inapplicability of patents to surgical procedures was first clarified during the mid-1840s when two American practitioners, Charles T. Jackson and William T.G. Morton, attempted to patent the use of sulphuric ether (later dubbed ‘Letheon’ by Morton) in surgical operations, much to the consternation of their medical brethren. On both sides of the Atlantic their actions brought to the fore questions not only about the practical deployment of patents but also about the ethical exceptionalism of surgical practice when it came to asserting ownership over new inventions. By first situating surgery within the broader context of nineteenth-century Britain's patent culture, I then go on to examine the alternatives to patenting that were used by surgeons to gain credit for their inventions. Surgery's lack of patentability did not preclude conversations and controversy over the different methods of claiming ownership that might be used. On the contrary, such issues loomed large within the medical press, which not only facilitated numerous priority disputes between medical men but often played an active role as unofficial arbitrator of priority claims. The example of operative surgery, I argue, also demonstrates the validity of examining fields of practice adjacent to those which endorsed patenting. What effect did it have on surgeons to live in a society which increasingly sought to formalize the protection of inventors’ ideas, and yet where many of their own inventions were not deemed patentable? Examining this issue expands our understanding of not only intellectual property in the medical realm, but also the nature of surgeons’ professional culture. Issues of priority, patenting and credit were critical to surgeons’ self-identity, at a time when their status was in the ascendance. Focusing on Britain, this chapter nonetheless speaks to the international context of cultures of credit, examining how the practices of American and French surgeons in this regard were understood by their British contemporaries.

## Humanity versus the sordid speculator: patenting ether inhalation

Surgeons of the late Victorian era believed the nineteenth century to have been the ‘surgical century’, and historians have noted that this was a period when the surgical profession underwent an elevation in its social status.^5^ Often this change in status has been connected to two specific occurrences: first the introduction of anaesthesia – ether and then chloroform – during the 1840s, and second the rolling out of antiseptic (and subsequently aseptic) measures during the last third of the century. Together, these two innovations have been seen to have vastly reduced the risks of surgery and, as a consequence, led to an expansion in the type of procedure that could be performed. The introduction of anaesthesia in particular has been singled out for the transformative effect it is thought to have had on surgical practice, leading to what historian Stephanie Snow has described as a ‘historical treatment of anaesthesia … disposed towards the celebratory’.^6^ In fact, as Martin Pernick has argued in his study of anaesthesia in nineteenth-century America, the number of operations performed was not greatly expanded by the introduction of ether, other than among the most gravely ill patients, while Snow has shown that in Britain ether was speedily replaced by chloroform.^7^

Nonetheless both acknowledge the immediate and powerful impact of the introduction of ether into American surgical practice and its rapid diffusion across the Atlantic. On 26 December 1846 *The Lancet* published a brief report of a speech given by Henry Jacob Bigelow, surgeon at the Massachusetts General hospital, detailing a set of operations that had taken place at the institution during which a method of procuring insensibility had been used. This method was the inhalation of sulphuric ether, already well known as a recreational drug that could cause unconsciousness. It had been administered by a dentist, William T.G. Morton, who claimed to have discovered the effects of ether in eradicating pain during dental extractions. Reports of Morton's experimental dentistry sufficiently impressed the hospital's surgical staff that he was invited to perform the procedure in conjunction with two operations at the hospital in October 1846. The first procedure, on a man requiring a jaw operation, was partially successful in mitigating the patient's pain. The second, on a woman with a large tumour on her arm, saw the patient profess to have been entirely pain-free and wholly insensible of her surroundings during the procedure.^8^ Together, the two operations were enough to convince Bigelow that he had witnessed a significant new discovery.^9^ By the time *The Lancet* had published his speech, news of the development had already spread among the British medical community.^10^ On 19 December the London-based dentist James Robinson extracted a tooth from a young woman made insensible by ether. Two days later Robert Liston, a surgeon at University College Hospital, successfully performed a thigh amputation under similar circumstances.

From its inception in Britain, ether inhalation was tightly bound up with questions of priority. *The Lancet*’s initial publication on the operations in Boston saw news of ether's wondrous effects somewhat checked by the rather earthlier matter of patenting which was already threatening to overshadow it. Taking up almost as much space as the description of the procedure itself, came the news that

Dr. Bigelow is reported to have patented the process on the grounds that such an agent is capable of abuse – that its action is not thoroughly understood – and because it is looked forward to as of especial use in dentistry, many of whose processes are secured by patent. Supposing the discovery to be genuine, even these offer but poor excuses for its reservation by patent.^11^

*The Lancet* had not interpreted Bigelow's speech quite correctly. His original report, published in the *Boston Medical and Surgical Journal*, in fact alluded to a patent taken out by Morton in partnership with Charles T. Jackson for the use of ether inhalation during operations. Jackson was a chemistry lecturer who had formerly taught Morton at medical school, where the two men had often discussed the possibility that sulphuric ether could procure a state of insensibility. Jackson had self-experimented with the substance a number of times, before Morton subsequently carried out a dental procedure on a patient under its influence in September 1846. The naming of Jackson as co-inventor had been the result of negotiations between the two men, and saw Jackson transfer his rights to Morton in exchange for the latter paying him a fixed percentage of any profits he might accrue. However, the two men would later enter into a disagreement, with Jackson claiming to be the sole ‘inventor’ of ether inhalation.^12^ Morton would also become embroiled in further disputes as other practitioners emerged to stake their claim, the highest-profile of whom was Horace Wells, Morton's former partner in dental practice, who claimed to have discovered a mode of insensibility for operations prior to Morton. The bitterness of the dispute and Wells's fate – he committed suicide in 1848, penniless and imprisoned, while under the influence of chloroform – served as a cautionary tale of the emotional investment of practitioners in their inventions, and the disappointment of those whose innovations failed to be recognized by their peers.^13^

The British journals played on the dichotomy apparent in the ether debate, pitching the moral qualities of the invention itself against the attempt to patent it. The humane objective of ether inhalation, to relieve the misery of pain, only served to highlight the unsavoury nature of claims to its personal ownership; and the idea of patenting within surgery, an arena of medicine fraught with physical torment, smacked of cruelty. Resistance against the patent was, the *Medical Times* decreed, the ‘forcible protest of humanity against the sordid speculator’, and the journal claimed that each operation on British soil was a victory against the mercantile quacks behind the patent.^14^ The controversy also provided an opportunity for British journals to remind their readers of the distinctly American nature of the scramble to patent, and the commercialism especial to American medical culture.^15^ American commentators would no doubt have disagreed with such a judgement: despite high-profile support of Morton from Bigelow, his early champion, the majority of practitioners made no bones about their distaste for the patent. As Joseph Gabriel has argued, in mid-century America, medical patents were roundly disapproved of by the profession as strongly as they were in Britain.^16^

Nonetheless it was quickly apparent that Morton was intent on having his rights upheld internationally, and a patent agent, James A. Dorr, was duly hired to represent his interests in London. In January 1847 Dorr wrote to the medical weeklies, using the correspondence columns of *The Lancet* and the *Medical Times* to announce that the use of ether inhalation in England and the colonies was patented and that those practitioners who used it would be liable to charges of infringement if they administered the substance without permission. Predicting in his letter that he would find an unfavourable response, Dorr was perhaps not surprised when his announcement was quickly brushed off on the grounds of impropriety.^17^ Patents of all kinds, whether they were for instruments, medicines or procedures, were seen as incompatible with the moral mission of the medical profession; they indicated a practitioners’ direct engagement with commercial activity, and thus a prioritization of self-interest over scientific progress and patient care. But it was also the impracticable nature of Morton and Jackson's patent that was singled out for criticism. This impracticability was bound up in the fact that Morton was attempting to patent the administration of ether rather than the drug itself. Although Morton had initially claimed the compound he used was not ether but an original composition called ‘Letheon’, it was widely known that they were one and the same. Thus the claim to novelty which the patent hinged upon was the use of ether as a method of procuring insensibility in surgical operations rather than the substance itself.^18^ The British press reacted with incredulity. To do so, the *Medical Times* commented, was like attempting to ‘bind the winds, not less absurd but improper’.^19^ Francis Boott, a London-based American physician whose house had been the location for Robinson's dental extraction under ether, drafted in a lawyer to comment on the patent and sent the ensuing correspondence to *The Lancet*. ‘An inventor may have a patent for the manufacture of particular medicinal preparations’, wrote the anonymous QC, ‘but who ever heard of a patent for the performance of a new operation in surgery (as, for example, that by which squinting is cured)? I can see no distinction in principle between such a patent and the patent supposed to be claimed for the administration of ether’.^20^

Thus, while both were morally dubious, the lawyer drew a legal distinction between patenting medicinal compounds and patenting the ether inhalation procedure, the latter of which he judged to be no different from attempting to patent the operation itself. His thoughts echoed the sentiments of many in the profession. If it was well known that Letheon was simply ether, then what was to stop a patient turning up with a bottle of it and administering it to themselves? Would a surgeon still then be liable to pay a fee for its use in the operation? How could any patent be enforced when, for those surgeons working charitably among the poor, fees were so scant anyway? British medical journals speculated upon the myriad difficulties that would lie ahead.^21^ They also repeated verbatim American dental surgeon Josiah Flagg's rallying call against Morton's patent: that patented ether was like ‘*patent sun-light* or *patent moon-shine*’.^22^ Flagg's turn of phrase has been interpreted as alluding to Morton's attempt to gain ownership of something that was construed by most as a universal right: freedom from pain.^23^ But his choice of words also suggested the slipperiness, the intangibility, of trying to establish ownership of an immaterial process; ‘what is patented?’ asked Flagg, in relation to Morton's claim, ‘A power? A principle? A natural effect? The operation of a well-known medicinal effect?’^24^ To most in the medical community it was simply unclear.

In America Morton would go on to try several more times to enforce his patent, culminating in an attempt in 1862 to recover infringement damages from the New York Eye Infirmary for their use of ether inhalation. But the case merely clarified the weakness of Morton's claim, which was subsequently voided on account of its lack of novelty.^25^ Nonetheless it was the distinction that the patent clarified between product and method which would be its lasting legacy: the ‘Morton doctrine’ continued to govern the non-applicability of medical method patents in the USA until relatively recently.^26^ Morton's claim soon disappeared from the British press too, and the patent does not appear to have been finalized in the United Kingdom.^27^ Nonetheless Morton, by means of Dorr, had opened up a significant point of debate. In April 1847 Dorr sent a long and impassioned letter to *The Lancet.* In what was one of the most open discussions of the topic to grace the journal's pages in the nineteenth century, Dorr implored the medical profession to reconsider their disapproval of patenting, or, at the very least, to look to an alternative system which made pecuniary rewards available to medical men. For Dorr, who had clearly detected the ambiguous, complex system of indirect credit and reward at the heart of the profession, rank and honour were hardly a replacement for financial compensation. After all, Dorr asked, ‘what is honour without the means of subsistence?’^28^ No reply was ever published.

## Surgery and cultures of credit

That patenting was, for both moral and practical reasons, seen as inapplicable to medicine and surgery did not quell the subject of reward for practitioners. On the contrary, it likely exacerbated the issue. After all, doctors had only to look beyond their profession to notice the disparity between rewards for medical inventions and those for other forms of innovation. As Dorr put it in his plea to the profession, why should not Morton have been ‘entitled to compensation as he who makes an improvement in the manufacture of woollen or other fabrics’?^29^ Dorr may have had a vested interest in the matter but, as the century progressed, the lack of pecuniary rewards for surgeons appeared increasingly discordant with trends in both surgery and in innovation more generally. In the middle decades there was growing recognition in Britain of the contributions made to society by inventors, embodied especially in engineers like Isambard Kingdom Brunel, George Stephenson and James Watt. This resulted in growing calls for inventions to be better recognized, legally and financially, and reinvigorated a lengthy campaign by manufacturers, inventors and other interested parties for wide-scale amendment to patent law, principally to increase the year-long tenure that patents could be held for, and also to reduce their initial price. The Patent Amendment Act, which fulfilled both these criteria, was passed in 1852.^30^ Meanwhile, in surgery, visible and dramatic innovations were taking place that were changing the landscape of practice.^31^ The introduction of abdominal surgery was heralded as a striking innovation, and was most clearly embodied in ovariotomy, a procedure to remove large non-malignant ovarian tumours, that had begun to be performed in Britain in the early nineteenth century and had become part of established practice by the 1860s.^32^

Although borne of innovations from a range of countries, ovariotomy was cherished as a British invention.^33^ Indeed, retaining British ownership of the operation became increasingly important in the face of French surgeons beginning to take up the operation in the 1860s. Looking to France exacerbated concerns in Britain about the relative paucity of rewards for surgeons. In France there was a long tradition of incentivising contributions to medicine and surgery through prizes and both the French Academy of Science and the French Academy of Medicine offered rewards for innovation. This included the prestigious Prix Barbier which had been established in 1846 with three sub-categories, one of which was specifically for the invention of instruments, operative techniques and surgical sundries, such as bandages.^34^ In 1863 Eugene Koeberlé, at that point one of very few surgeons who performed ovariotomy in France, was awarded the prize, worth two thousand francs, by the Academy of Medicine for having performed two successful ovariotomies; this despite Koeberlé being, in the view of the *British Medical Journal*, ‘merely a copyist of the English in the matter of ovariotomy’.^35^ While some medical prizes did exist in Britain, dispensed through institutions like the Royal College of Surgeons of England, they were sparse, and their pecuniary value was considered paltry in comparison to those given out by institutions across the channel.^36^ The prospect of state reward was also unlikely. While there had been a relatively well-established system of parliamentary reward for scientific and technical innovation in Britain during the eighteenth and early nineteenth centuries, the best-known medical recipient of which had been Edward Jenner, the system had all but disappeared by the middle decades.^37^ In 1872 the physician and journalist Andrew Wynter, in an essay for the *Edinburgh Review* on the progress of medicine and surgery during the last fifty years, reflected on the medical profession's thankless pursuit of innovation. Singling out chloroform anaesthetic and ovariotomy as key examples of British industry, Wynter protested that ‘some tangible evidence should be given that the nation appreciates the sacrifices daily and hourly made by those who devote their energies and their talents to the promotion of its physical well-being’.^38^ Wynter's protestations, like Dorr's, appear to have fallen on deaf ears.

In 1867 the Birmingham surgeon Sampson Gamgee set off on a two-week holiday to Paris in which he took in both the World Fair and the state of surgery in the city. Gamgee wrote to *The Lancet* about the trip; ‘many are crowding to Paris, and wondering at the progress made by the French nation in a variety of manufacturing and industrial departments, in which, not many years ago, we enjoyed a clear, and scarcely questioned, supremacy’, he observed.^39^ His investigation of French surgery was likewise infused with the language of comparison as he diplomatically negotiated his way through similar and contrasting aspects of French and British surgical practice. Gamgee depicted French surgeons as organized and well educated – indeed perhaps too well educated – at the cost of their practical abilities. British surgeons, on the other hand, he viewed as practically minded doers, who were more fearless as operators. Gamgee stretched out this analogy to British industry as a whole:

The engine-driver on a French railway is often a good pupil of the École des Arts et Métiers, knows a great deal about physics, and every now and then is nearly as good a mathematician as he is a mechanic; but he would be sorely puzzled to match one of our men in piloting the Holyhead mail at fifty miles an hour through a November fog.^40^

It was these uniquely British characteristics of courage, practicality, persistence and boldness that for Gamgee both defined British surgeons and engineers and enabled them to retain their standing even in the face of national competition. And yet, while it became increasingly acceptable for engineers to patent their inventions, relatively free of accusations of moral impropriety, surgeons were bereft of official channels through which to make similar claims. For the surgical community it meant that claims to ownership had to be legitimated through other means of authority, of a specifically medical kind.

## Different modes, different plans: publishing and priority disputes

In 1837 *The Lancet* ran an editorial about doctors’ excessive interest in credit and priority. The journal complained,

The extent to which this evil has grown can only be fully appreciated by the conductors of the periodical press, or by those who follow with attention the debates of our medical and philosophical societies. Editors’ tables are continually laden with letters from gentlemen, who would enforce their claim to ‘priority’ in some discovery.^41^

There was an irony to *The Lancet*’s words: the emergence of the weekly medical press, with the significant space it set out for correspondence and subsequent speedy responses, made it fertile ground for disputes that had no means of being satisfied through other channels like patenting. While, ostensibly, editors like *The Lancet*’s Thomas Wakley did not approve of this trend, this certainly did not preclude them from publishing such disputes and, at times, seemingly encouraging them. Nor did possible associations with unsavoury self-interest stop practitioners airing their grievances publicly. As we have seen with the example of ether, it was through journals that these claims and disagreements were actively shaped and mediated. The growing importance of the medical periodical as a tool of professional cohesiveness meant that not only did they facilitate debates but, as self-declared upholders of professional ethics, they often acted as judges when claims were disputed.^42^ In the following I show how claims to surgical innovation functioned through channels alternative to patenting: through interwoven strategies of eponymy, publishing and publicized disputes in the correspondence columns of medical journals. I purposely employ the term ‘credit’ to consider them. This is not only because that was the term frequently used during discussions of priority, but also because it conveys the somewhat obscure relationship between honour, attribution and financial profit underpinning them.

Attributing credit to surgeons, both past and present, can be complicated. Medical sociologists Judith P. Swazey and Renée C. Fox have argued that this is because multiple types of priority hover around surgical innovation. Priority for performing a new operation for the first time may differentiate from priority for performing a new operation successfully for the first time, or priority for being the first to publish on it.^43^ Building on Swazey and Fox's argument, a close examination of the way surgical priority played out in the nineteenth century suggests that credit claims could in fact be even more multifaceted than they suggest. Surgeons frequently wrote in not only to claim the first performance of a new operation, but also to claim new modifications on an existing procedure or for an instrument designed to complement a new operation.^44^

**Figure 1.**
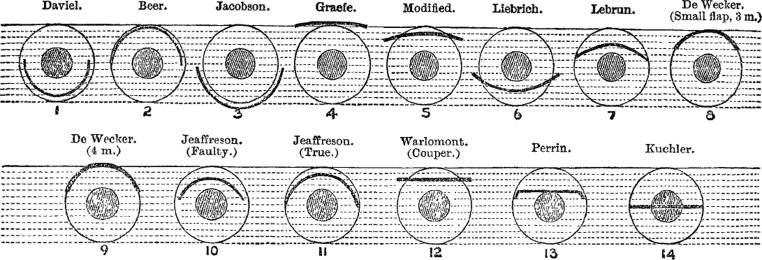
C.S. Jeaffreson's illustration of fourteen plans for operating in cataract surgery. C.S. Jeaffreson, ‘Clinical lecture on cataract’, *The Lancet* (1886) 127(3262), pp. 434–437, 436.

Indeed, it was the constant modification and lack of standardization in surgery that could make disputes over credit particularly perplexing, as surgeons developed and changed procedures, often while in the middle of surgery.^45^ This was frequently followed by an eager letter to a journal describing a new ‘mode’ or ‘plan’ for operating. That was certainly the case with one of the oldest procedures in surgery, the removal of cataracts. Cataract surgery in its modern form had originated from France in the mid-1700s, but over the nineteenth century it was subject to numerous modifications by surgeons across Europe, with flurries of activity and innovation occurring periodically. ‘Perhaps no operative procedure in the whole range of surgery offers such an *embarras de richesse* as that of removing a cataract’, commented Newcastle surgeon Christopher Jeaffreson in 1886. He went on, ‘the names and inventors of cataract operations and cataract instruments would, I verily believe, fill a column of *The Times* newspaper, and some of these inventors, I am uncharitable enough to think, would be well pleased to see such a list in print’.^46^ Jeaffreson's observation appeared to allude to a yearning among ophthalmic surgeons to have their contributions publicly recognized in what was a crowded market for innovation. Jeaffreson counted no less than fourteen modes to the operation, each using a different type of incision, which he grouped together in an accompanying illustration (see Figure 1).

As the image conveyed, connecting one's name to a particular technique was one obvious route through which to ensure a close degree of association with a new procedure, and eponymy was often employed in relation to both operations and instruments, although probably more so with the latter. As Claire L. Jones has shown, in the case of instruments and devices, eponymy was viewed as a legitimate, morally acceptable way to claim ownership. Most surgeons who invented or modified devices freely gave their designs to manufacturers and received no direct financial compensation. But as Jones highlights, eponymous instruments could enhance the reputation of both manufacturers and medical men. In surgical trade catalogues, the close associations between individual surgeons and their instruments could be an important promotional technique.^47^ This speaks to the broader symbiotic relationship between surgeons and ‘their’ instruments: the most popular instruments tended to be those made by high-status surgeons, whose names suggested the trustworthiness of the tool. In ovariotomy, for example, the instruments devised by the most famous practitioner of the operation, Thomas Spencer Wells, were the most highly regarded, and Wells was particularly good at maintaining his profile in the field with numerous instruments of his design.^48^ The popularity of such instruments could then work to further reinforce a surgeon's name and reputation, which in turn likely increased the possibilities for lucrative private practice. However, because financial reward and eponymous recognition remained indirectly connected, this enabled surgeons to frame their use of eponymy over patenting as a matter of pure altruism. A retrospective article from the lay press in 1949, on the prolific American surgeon James Marion Sims, who had numerous instruments named for him (such as Sims's speculum, and the Sims catheter) recalled, ‘Another distinguishing feature in his life was his utter unselfishness. Millions of dollars would have flowed simply by his retaining rights on his numerous inventions of surgical instruments. He refused all monetary returns; he gave them freely, he took out no patents.’^49^ Sims would, however, build up a large private practice of wealthy patients across Europe and America during the middle decades of the century.

Nonetheless eponymy alone could not be counted upon for ensuring and maintaining credit. For surgeons, publishing was also becoming essential to establishing claims. Turning back to ovariotomy, the operation provides an interesting example of how this could play out. As has been described above, the operation had been in practice in Britain since the late 1830s, although only accepted by the majority of the profession during the 1860s. One of the most prolific performers of the operation in its early days had been Manchester obstetrician Charles Clay, who had performed his first ovariotomy in 1842 and who had also been the first surgeon to enlarge the abdominal opening from a ‘minor’ incision of a few inches to a ‘major’ one of around twelve. Clay, along with other early practitioners of the procedure, had been subject to many derogatory comments in the early days of the operation, those opposing ovariotomy declaring Clay and his contemporaries ‘belly-rippers’ for venturing into the abdomen. Although a successful operation could result in a complete cure, it was also a dangerous procedure and around a third of patients died during it or soon after. Thus by no means all were convinced that the risk of the operation was worth taking.

When the operation did begin to garner widespread support it would not, however, be Clay's name most closely associated with its transition in status, but another man's, the aforementioned Thomas Spencer Wells. In the 1860s Wells had also become a regular practitioner of the operation some twenty years after Clay had begun, and in 1865 had published a book, *Diseases of the Ovaries: Their Diagnosis and Treatment*. The book was quickly regarded as influential. But Wells's success was less to do with his mortality rate – which at around one-third was about the same as Clay's – but rather with the way he represented his cases. Clear, concise statistics recounting the hundreds of patients who had come under his care, as well as detailed notes for each, the book was an unprecedented collection of published material on the operation. Seventeen years younger than Clay, Wells's understanding of the necessity of publishing to secure credit had been shaped by a rapidly expanding world of medical print culture. Clay, on the other hand, had never seen fit to publish all of his operations. The result was a bitter dispute between the two men that began around the time Wells published his book. Wells wrote,

Half a page of tabulated matter is really all the information published of 50 of Dr. Clay's alleged cases, except some equally useless lists in one of Dr. R Lee's tables. Such meagre unauthenticated reports are absolutely worthless to the scientific inquirer; and, for all purposes of comparison with the results of other operators, Dr. Clay can only be admitted as having operated on 27 patients.^50^

For Wells, then, despite Clay's assertion that he had performed the operation 111 times, only his twenty-seven published case reports counted. The onus was increasingly upon surgeons to publish to ensure that the rest of the surgical community could also, in a sense, witness their operations. This was viewed as particularly important in the field of ovariotomy, where fears had long abounded that failed cases were going unreported.^51^ Wells's refusal to adequately acknowledge Clay led to the two practitioners offering very different interpretations of how credit should be defined. Wells indicated that cases had to be connected together in a series, preferably as a monograph, to bring clarity to one's results, and thus ascertain credit. But for Clay, credit was constructed differently and more closely bound to originality and priority.^52^ For him, the fact that he performed the first successful ovariotomy by major incision in England, and then performed it consistently, was enough to define him as the first credible ovariotomist in England. ‘If I had not been the pioneer for this operation in 1842, and for years after that, alone and unsupported,’ Clay would later claim, ‘neither ovariotomy as an operation, nor Mr. Wells as an ovariotomist, would most probably be heard of at this time.’^53^ His words evoked a romantic image of the isolated inventor who had risked his reputation in the name of progress.^54^ Clay also emphasized the idiosyncratic nature of surgery, at times suggesting a polarity in his and Wells's methods, and thus intimating that their operations were entirely different from one another.^55^

Ultimately Wells's claim to ovariotomy won out, buttressed by his impressive publication record. Clay's reputation, on the other hand, was severely damaged when he accused Wells of performing unnecessary and risky operations in pursuit of large fees, the Manchester practitioner having made the mistake of crossing the highly policed, albeit paper-thin, line between questions of credit and questions of commerce.^56^ In 1880 the controversy had been once more revived when the *British Medical Journal* published an editorial celebrating Wells's thousandth ovariotomy and commending the surgeon for establishing the operation. Clay wrote furious letters to the journal, demanding that, as it seemed to view itself ‘a judge of equity in professional matters … you ought to be the better able to act fairly in this matter’, thus positing the journal itself as arbiter.^57^ The journal nonetheless maintained that credit for ovariotomy was rightfully Wells's, who would reap the rewards in real terms, and become a substantially richer man than his rival. In what could only have been an allusion to Wells, who had become a surgeon to the Royal Household in 1863 and was created a baronet in 1883, Clay was in the habit of telling friends near the end of his life that ‘some men have got baronetcies, some wealth, some positions at Court, but I have got peace of mind’.^58^

The road to credit and priority could be difficult for surgeons of the nineteenth century. Claims to credit had to be negotiated with care, and within the closely policed boundaries of medical etiquette. The benefits of establishing a personal connection with a successful innovation were clear: intellectual credit could transform into social and financial credit. Nonetheless, as the case of Charles Clay demonstrates, such claims were rarely fully secure. Rather, they had to be constantly maintained in the face of subsequent competitors who could potentially overshadow earlier contributions.

## Conclusion

The essays in this special issue speak to a growing interest among historians in the role patenting has played in medicine. In comparison to the history of science, however, it can still feel like the history of medicine has some way to go in examining the antecedents of intellectual property today. The relative lack of overt engagement that ‘orthodox’ practitioners had with patenting in the nineteenth century has perhaps led to a misleading assumption that the connection does not exist at all. Thinking about the way surgeons formulated models of intellectual ownership suggests a new way of moving forward, of thinking beyond patenting, and towards other forms of priority and credit that abounded. Social credit – with the hope of future financial reward – was hard-won but easily lost by surgeons. That this credit was unofficial made it a sometimes perilous and often failed pursuit. The process of securing credit could be lengthy, delayed and convoluted. Nonetheless, if one was successful, the rewards could be bountiful; these accolades made the risks of innovation worthwhile.

Looking at how priority and credit functioned in surgical practice is also a way of enriching the history of surgery. Far from being a humorous or mildly unsavoury aside in the biographical narratives of eminent surgeons, questions of credit and priority should be understood now as they were in the nineteenth century: as central to professional surgical culture and closely connected to surgeons’ group identity. By the late nineteenth century, surgeons were no longer inferior in social status to physicians. On the contrary, they had risen above their counterparts. Managing claims to credit helped surgeons make sense of the innovations of their era and themselves as agents of historically significant change and improvement.^59^ By examining this we move closer to understanding the complex notions of what constitutes surgical knowledge and the implications of surgery's physicality on claims to intellectual property in the field.

## Footnotes

1 Intellectual Property Office, *Examination Guidelines for Patent Applications Relating to Medical Inventions in the Intellectual Property Office* (May 2013), p. 6.

2 This framework was legally validated in 1996 following the case of *Pallin* v. *Singer.* In 1992 ophthalmic surgeon Dr Samuel Pallin patented an incision technique for cataract surgery. The following year he attempted to sue another ophthalmic surgeon for using the technique without a license. Pallin's infringement claim was eventually invalidated and led to a powerful lobby from the surgical profession to prevent practitioners from patenting surgical methods. That same year Congress passed a bill, something of a compromise, which continued to allow patenting of medical and surgical processes but banned any patentee from seeking to recover damages from patent infringement. Katherine J. Strandburg, ‘Legal but unacceptable: *Pallin v Singer* and physician patenting norms’, in Rochelle Cooper Dreyfuss and Jane C. Ginsburg (eds.), Intellectual Property at the Edge: The Contested Contours of IP, Cambridge: Cambridge University Press, 2014, pp. 321–342; NYU law and economics research paper, Bo. 14-42, available at http://papers.ssrn.com/sol3/papers.cfm?abstract_id=2538557, accessed 29 April 2015.

3 AsifE, ‘Exclusion of diagnostic, therapeutic and surgical methods from patentability’, Journal of Intellectual Property Rights (2013) 18(3), pp. 242–250.

4 *Myriad Genetics* v. *Association of Molecular Pathology*, No. 12-398 (569 U.S. 13 June 2013). In 2013 the Supreme Court invalidated the patents that Myriad Genetics, a diagnostic company, held on naturally occurring isolated gene sequences, although it upheld those for synthesized complementary DNA (cDNA). The case, which came to the District Court in 2010 and has been through numerous appeals, has highlighted concerns regarding the commercial nature of the rapidly expanding field of personalized medicine.

5 In 1902 Leeds surgeon Arthur Mayo Robson lauded the work of Joseph Lister, ‘whose name is one of the brightest ornaments of the nineteenth century and whose work will make that era stand out for all time as the surgical century’. ‘An introductory address on the advance in surgery during 30 years’, The Lancet (1902) 160(4127), pp. 912–916, 913. On the status of surgeons in the nineteenth century see Christopher Lawrence, ‘Democratic, divine and heroic: the history and historiography of surgery’, in Lawrence (ed.), Medical Theory, Surgical Practice: Studies in the History of Surgery, London: Routledge, 1992, pp. 1–47.

6 Stephanie Snow, Operations without Pain: The Practice and Science of Anaesthesia in Victorian Britain, Basingstoke: Palgrave Macmillan, 2006, p. 2.

7 Martin S. Pernick, A Calculus of Suffering: Pain, Professionalism, and Anesthesia in Nineteenth-Century America, New York: Columbia University Press, 1985, p. 216; Snow, op. cit. (6), p. 67. Not only was ether difficult to administer, but Snow describes how inhalation often induced ‘excitement rather than insensibility’ in patients, making operations difficult to perform.

8 Henry Jacob Bigelow, ‘Insensibility during surgical operations produced by inhalation’, Boston Medical and Surgical Journal (1846) 35(16), pp. 309–317, 309.

9 Bigelow, op. cit. (8), p. 317.

10 Snow, op. cit. (6) p. 41.

11 ‘Medical news’, The Lancet (1846) 48(1217), p. 704.

12 Martin Gay, A Statement of the Claims of Charles T. Jackson, M.D to the Discovery of the Applicability of Sulphuric Ether to the Prevention of Pain in Surgical Operations, Boston: David Clapp, 1847.

13 ‘Death of Dr Horace Wells’, *Boston Medical and Surgical Journal* (1848) 38(1), pp. 24–25. Prior to Morton, Wells had performed a number of dental extractions where he had used nitrous oxide to successfully eradicate pain. Nonetheless a public demonstration of his method at Harvard medical school in 1845 failed, humiliating Wells in the process and permanently damaging his career. Despite this, Wells continued to maintain that he had discovered the principle of insensibility in operations before Morton. Sarah H. Gordon, ‘Wells, Horace’, in *American National Biography Online*, available at www.anb.org/articles/12/12-00962.html, accessed 27 May 2016.

14 ‘To correspondents’, Medical Times (1847) 15(381), pp. 306–307, 307.

15 Robert Frankel cites the somewhat unfavourable accounts of America written by British authors in the 1830s and 1840s. This included Charles Dickens's *American Notes for General Circulation*, in which Dickens criticized the commercial nature of American culture and ‘the national love of trade’. Robert Frankel, Observing America: The Commentary of British Visitors to the United States, Madison: University of Wisconsin Press, 2007, p. 9; Charles Dickens, American Notes for General Circulation, 2 vols., London: Chapman and Hall, 1842, vol. 2, p. 292.

16 Indeed, arguably even more so. As Joseph M. Gabriel has shown, in 1847 the American Medical Association's first code of ethics explicitly stated that patenting of medicines and surgical instruments was contrary to professional ethics. Joseph M. Gabriel, ‘A thing patented is a thing divulged: Francis E. Stewart, George S. Davis, and the legitimization of intellectual property rights in pharmaceutical manufacturing, 1879–1911’, Journal of the History of Medicine and Allied Sciences (2009) 64(2), pp. 135–172, 1431915141910.1093/jhmas/jrn075. For a discussion of the patented-ether debate in the US and its implications for medical morality and patenting culture see also Joseph M. Gabriel, Medical Monopoly: Intellectual Property Rights and the Origins of the Modern Pharmaceutical Industry, Chicago: The University of Chicago Press, 2014, pp. 60–63.

17 Dorr wrote, ‘I am aware that doubts exist in the minds of some as to the liberality of rendering inventions or improvements, which tend to alleviate suffering, subjects of patents.’ James A. Dorr, ‘Surgical operations performed during insensibility, produced by the inhalation of sulphuric ether’, The Lancet (1847) 49(1218), p. 8; Dorr, ‘Advertisement’, *Medical Times* (1847) 15(379), p. 254.

18 Their patent specified that it was the method of combining ether with – or applying it to – a surgical operation that was being patented. Charles T. Jackson and William T.G. Morton, ‘Improvement in surgical operations: specification forming part of letters patent no. 4,819’, United States Patent Office, 1846.

19 ‘To correspondents’, Medical Times (1847) 15(381), p. 307.

20 Francis Boott, ‘Inhalation of ether in surgical operations’, The Lancet (1847) 49(1219), pp. 49–50. The example of surgery for squinting was not indiscriminate. In the early 1840s a new operation for curing squinting by sectioning of the media rectus muscle had been developed by Berlin surgeon Johan Dieffenbach (1792–1847) and the operation had rapidly diffused across Europe. Thus it served as a relevant, comparative example in the field of operative surgery.

21 As the *London Medical Gazette* put it bluntly, ‘it is clear, therefore, that the patentees, if successful in their object, can look for a satisfactory return only to the legs and arms of the wealthy part of the community’. ‘[Leading article]’, *London Medical Gazette* (1847) 4, pp. 66–70, 69.

22 Josiah Flagg ‘The inhalation of an ethereal vapor to prevent sensibility to pain during surgical operations’, Boston Medical and Surgical Journal (1846) 35(18), pp. 356–359, 357, original emphasis.

23 Gabriel, op. cit. (16), p. 61.

24 Flagg, op. cit. (22), p. 357.

25 Samuel Fisher, Reports of Cases Arising upon Letters Patent for Inventions Determined in the Circuit Courts of the United States, Cincinnati: Robert Clarke & Co, 1871, vol. 2, pp. 320–330.

26 Asif E, op. cit. (3), p. 246. The author notes the use of the Morton case as judicial doctrine well into the twentieth century. It was superseded by the 1996 *Pallin* v. *Singer* case.

27 There is no record of such a patent in the United Kingdom Patent Office's 1852 reference index.

28 James A. Dorr, ‘Are improvements in medicine and surgery proper subjects of patents?’, The Lancet (1847) 49(1237), pp. 523–524.

29 Dorr, ‘Surgical operations performed during insensibility’, op. cit. (17), p. 8.

30 Christine MacLeod, Heroes of Invention: Technology, Liberalism and British Identity, 1750–1914, Cambridge: Cambridge University Press, 2007, pp. 2–3. For an overview of patent law in the nineteenth century see H.I. Dutton, The Patent System and Inventive Activity during the Industrial Revolution, 1750–1852, Manchester: Manchester University Press, 1984, pp. 34–56.

31 Anne Digby has suggested that it was not necessarily the case that there was more innovation in surgery compared to medicine, but that it was more visible and striking. Anne Digby, Making a Medical Living: Doctors and Patients in the English Market for Medicine, 1720–1911, Cambridge: Cambridge University Press, 1994, p. 96.

32 Sally Frampton, ‘“The most startling innovation”: ovarian surgery in Britain, *c.*1740–1939’, unpublished PhD dissertation, University College London, 2014.

33 The possibility of removing diseased ovaries had first been suggested by French practitioners in the early eighteenth century but the operation had not been put into practice. The first successful performance of the procedure is generally credited to the American surgeon Ephraim McDowell, who undertook the operation in 1809. Nonetheless British surgeons believed it was they who should be credited as the chief innovators of ovariotomy as it was they who had established its propriety and success through consistent practice. See Frampton, op. cit. (32).

34 The prize was in fact established by Baron Joseph-Athanase Barbier, a surgeon.

35 George Weisz, The Medical Mandarins: The French Academy of Medicine in the Nineteenth and Early Twentieth Centuries, Oxford: Oxford University Press, 1995, pp. 98–103; anon., ‘Le Prix Barbier ses métamorphoses’, *Revue de thérapeutique Medico-Chirurgicale* (1861) 2(21), pp. 561–563, 562.

36 The closest equivalent in Britain was the Jacksonian Prize, founded in 1800 and given annually by the Royal College of Surgeons of England to reward essays written on pre-picked topics by members and fellows of the college. While some winners went on to become high-profile practitioners, such as Henry Gray, author of *Gray's Anatomy*, the surgical community's response to the prize throughout the century was lukewarm, perhaps because it required a written contribution rather than a technical one. A retrospective on the prize published in 1899 noted a lack of interest among members, with many years seeing no or hardly any entries. The pecuniary reward of ten to twelve pounds was also viewed as relatively low. ‘The Jacksonian Prize: a retrospect’, *British Medical Journal* (1899) 1(1996), pp. 818–820.

37 Robert Burrell and Catherine Kelly, ‘Public rewards and innovation policy: lessons from the eighteenth and early nineteenth centuries’, Modern Law Review (2014) 77(6), pp. 858–887.

38 Andrew Wynter, ‘Review essay’, Edinburgh Review, Or Critical Journal (1872) 136(278), pp. 488–515, 515.

39 Sampson Gamgee, ‘The present state of surgery in Paris’, The Lancet (1867) 90(2296), pp. 273–274, 273. While the prospect of increased competition from Germany and America loomed over Britain, Graeme Gooday has critiqued oversimplified characterizations of the 1860s as a period of rapid industrial decline in Britain. Graeme Gooday, ‘Lies, damned lies and declinism: Lyon Playfair, the Paris 1867 Exhibition and the contested rhetorics of scientific education and industrial performance’, in Ian Inkster (ed.), The Golden Age: Essays in British Social and Economic History, 1850–1870, Aldershot: Ashgate, 2000, pp. 105–120, 120.

40 Gamgee, op. cit. (39), p. 802.

41 ‘Editorial’, The Lancet (1837) 28(726), pp. 669–670, 670.

42 Michael Brown, ‘Medicine, reform and the “end” of charity in early nineteenth-century England’, English Historical Review (2009) 124(511), pp. 1353–1388.

43 Judith P. Swazey and Renée C. Fox, ‘The clinical moratorium’, in Renée C. Fox (ed.), Essays in Medical Sociology: Journeys into the Fields, New York: Transaction Publishers, 1988, pp. 325–365, 337.

44 For example, in 1866 the invention of a new instrument for use in ovariotomy, the adhesion clam, sparked a tense exchange between surgeons John Clay (no relation to Charles Clay) and Isaac Baker Brown. Clay's claim to this innovation initially seemed secure, he having invented the instrument and made both the details and design of it accessible by publishing them in the *Medical Times* in 1862. But in 1866 the instrument was used by Isaac Baker Brown to develop a new method for performing ovariotomy. Through the press Brown sought ‘due credit’ for being part of the process of innovation and for being the first to use the instrument to create a new mode of operating, a claim Clay disputed. John Clay, ‘Adhesion clam: a new instrument for aiding the removal of ovarian tumours etc’, Medical Times and Gazette (1862) 1, pp. 640–641; Isaac B. Brown, ‘Management of the pedicle in ovariotomy’, British Medical Journal (1866) 2(302), p. 421; John Clay, ‘On management of the pedicle in ovariotomy’, British Medical Journal (1866) 2(303), pp. 449–450. Frampton, op. cit. (32), pp. 149–153.

45 Sally Wilde and Geoffrey Hirst, ‘Learning from mistakes: early twentieth-century surgical practice’, Journal of the History of Medicine and Allied Sciences (2009) 64(1), pp. 38–771866957310.1093/jhmas/jrn055.

46 C.S. Jeaffreson, ‘Clinical lecture on cataract’, The Lancet (1886) 127(3262), pp. 434–437, 435.

47 Claire L. Jones, The Medical Trade Catalogue in Britain, 1870–1914, London: Pickering & Chatto, 2013, pp. 147–148.

48 Indeed, even today Wells's artery forceps can be found in operating theatres.

49 ‘Ceremony in Lancaster to pay honor to Dr. J. Marion Sims’, clipping from the *Charlotte Observer*, 29 September 1949, University of North Carolina at Chapel Hill, J. Marion Sims Papers, 1835–1884, 00666/6.

50 T. Spencer Wells, ‘Results of ovariotomy’, The Lancet (1865) 85(2167), p. 272.

51 ‘Royal Medical and Chirurgical Society, November 13th 1850’, The Lancet (1850) 56(1421), pp. 583–587.

52 Sally Frampton, ‘Patents, priority disputes and the value of credit: towards a history (and pre-history) of intellectual property in medicine’, Medical History (2011) 55(3), pp. 319–324, 3222179225410.1017/s0025727300005330PMC3143866.

53 Clay, op. cit. (44), p. 201.

54 Frampton, op. cit. (52), p. 322.

55 Charles Clay, ‘On ovariotomy and ovariotomists’, The Lancet (1865) 85(2166), pp. 226–228, 227; Frampton, op. cit. (52), p. 323.

56 ‘Freedom V License’, British Medical Journal (1865) 2(259), pp. 637–638.

57 Charles Clay, ‘The history of ovariotomy’, The Lancet (1880) 2(1020), pp. 109–110, 110.

58 ‘Charles Clay: obituary’, The Lancet (1893) 142(3657), pp. 845–846, 846.

59 Frampton, op. cit. (52), p. 323.

